# Innovations in pain management in patients with chronic pancreatitis

**DOI:** 10.1016/j.igie.2025.03.005

**Published:** 2025-03-30

**Authors:** Sarah Enslin, Benjamin Bick, Sumeet K. Tewani, Vivek Kaul

**Affiliations:** 1 Division of Gastroenterology and Hepatology, University of Rochester Medical Center, Rochester, New York; 2 Rockford Gastroenterology Associates, LTD, Rockford, Illinois, USA

Abdominal pain is the most common and debilitating symptom of chronic pancreatitis (CP), affecting approximately 75% of patients at the time of diagnosis.^1^ This pain is often multifactorial, arising from mechanisms such as recurrent or chronic inflammation, ductal obstruction, neurogenic pathways, and centralized sensitized pain states.^2^ The complexity of CP-related pain poses significant challenges in management, requiring a multimodal approach that addresses both physical and mental health aspects while aiming to improve overall quality of life (Fig. 1).^3^^,^^4^ Ideally, this care is delivered in specialized multidisciplinary settings, where advanced practice providers (APPs) play a pivotal role.

**Figure 1:**
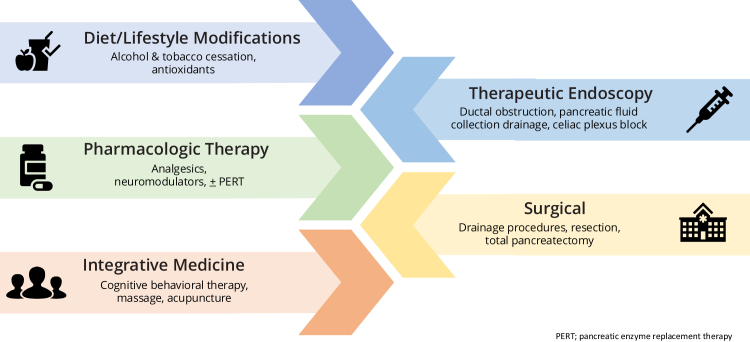
Multimodal strategies for pain management in chronic pancreatitis.

Over the past decade, the integration of APPs into subspecialty gastroenterology clinics has expanded, with APPs becoming key contributors to the management of complex gastrointestinal diseases such as CP. Equipped with specialized skills honed through mentorship and training, APPs provide continuity of care, actively participate in multidisciplinary discussions, and deliver tailored patient education (Fig. 2). In the management of CP, they leverage a variety of evidence-based strategies—including lifestyle modifications, pharmacologic interventions, integrative medicine, and procedural options—to effectively address the complexities of CP-related abdominal pain.

**Figure 2:**
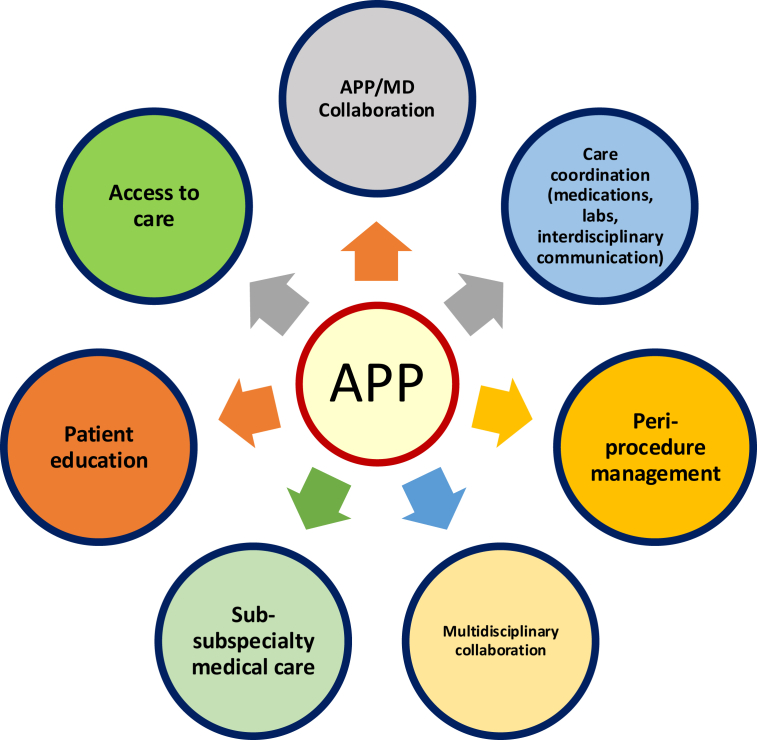
Role of gastroenterology advanced practice providers (APPs) in complex disease management. *MD*, medical doctor.

This article offers a concise overview of CP pain pathways and outlines a spectrum of management strategies (Fig. 3). Additionally, it highlights actionable insights into how APPs can expand their roles in multidisciplinary settings, enhancing patient care and potentially improving outcomes in this challenging patient population.

**Figure 3:**
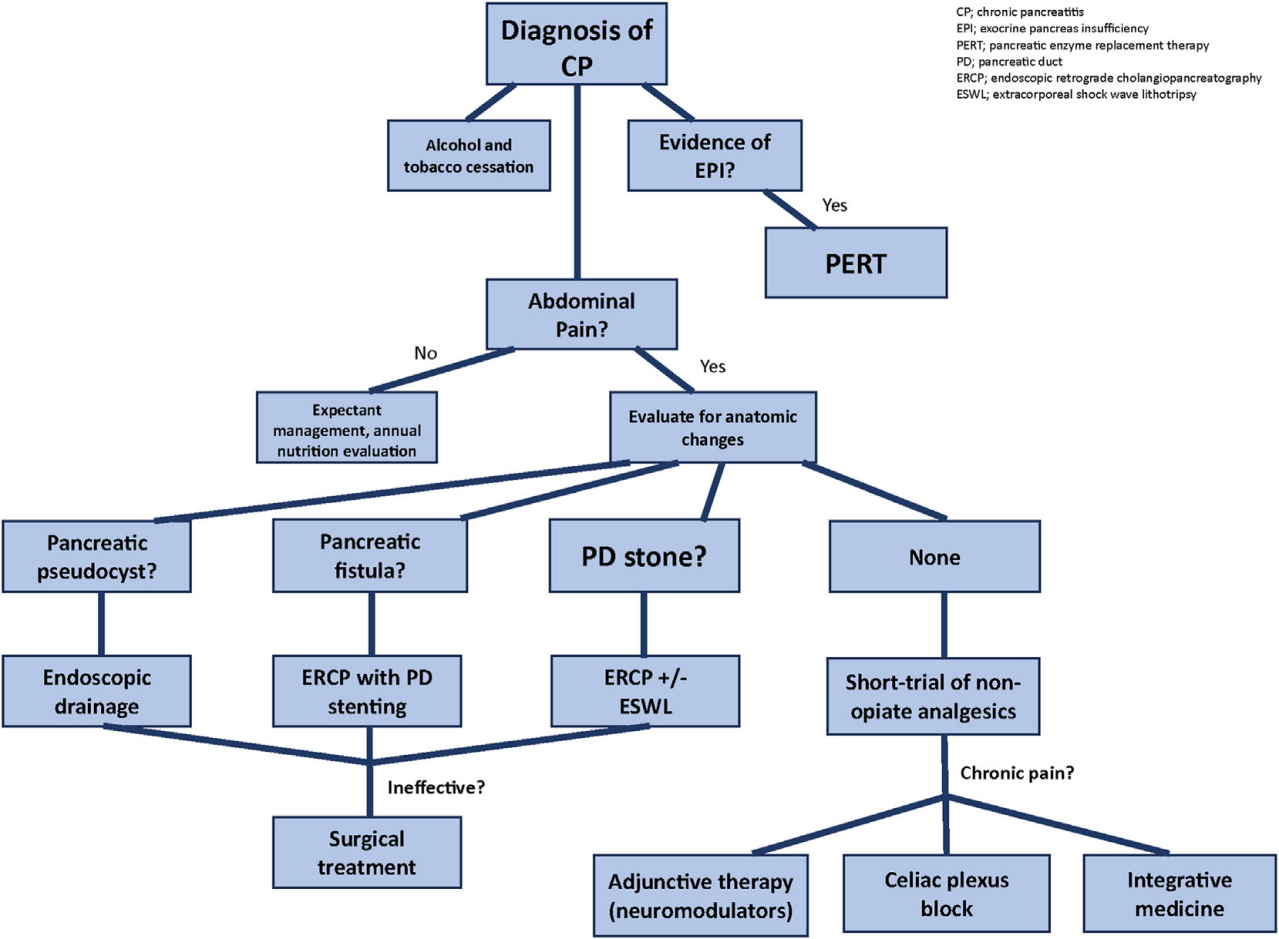
Proposed algorithm for pain management in chronic pancreatitis.

## Pain pathways in CP

Abdominal pain in CP may be described as a dull, aching, or sharp epigastric discomfort. It may radiate to the back and worsen after oral intake. The frequency, severity, and duration of pain vary widely and are often worse in patients with early-onset disease and alcohol-related etiology.^2^ Chronic and/or recurrent inflammation is a primary driver of pain in patients with CP.

**Figure 4:**
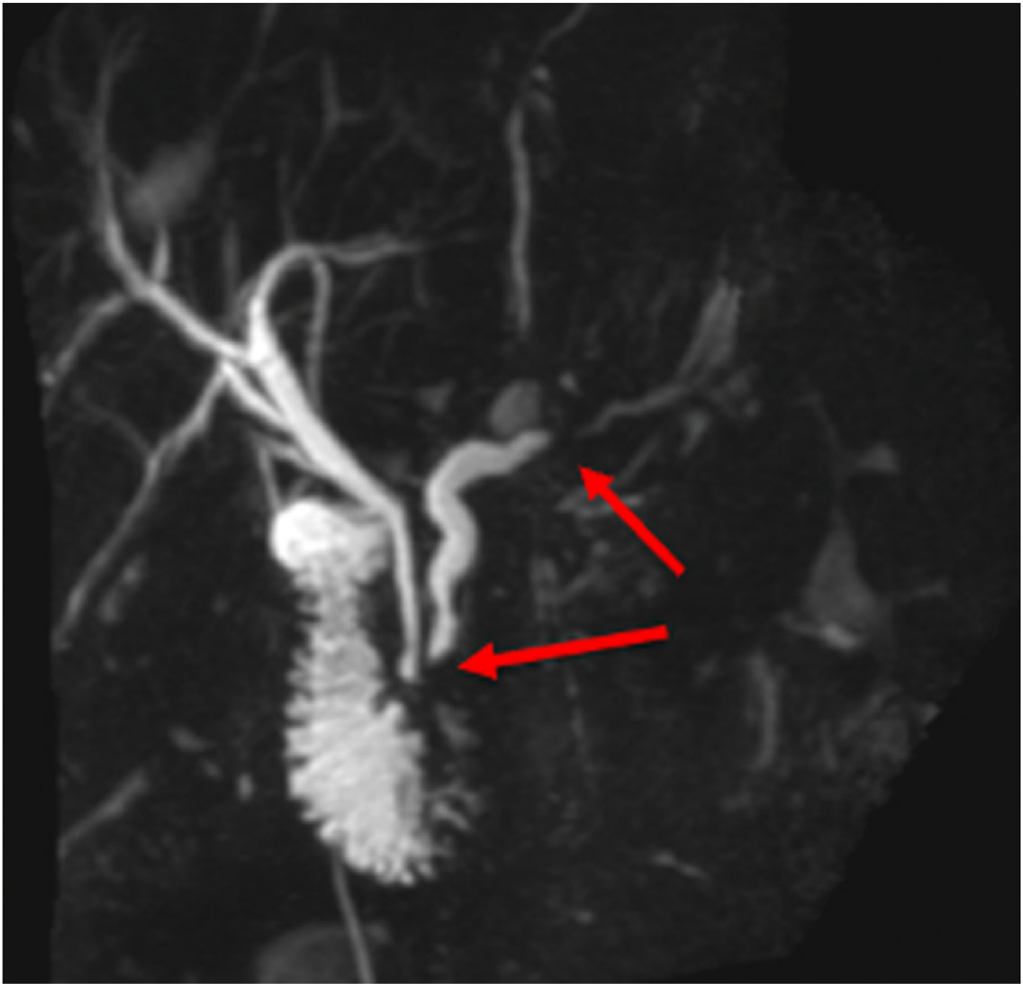
Magnetic resonance cholangiopancreatography image showing pancreatic ductal obstructions (*arrows*) in a patient with chronic pancreatitis.

**Figure 5:**
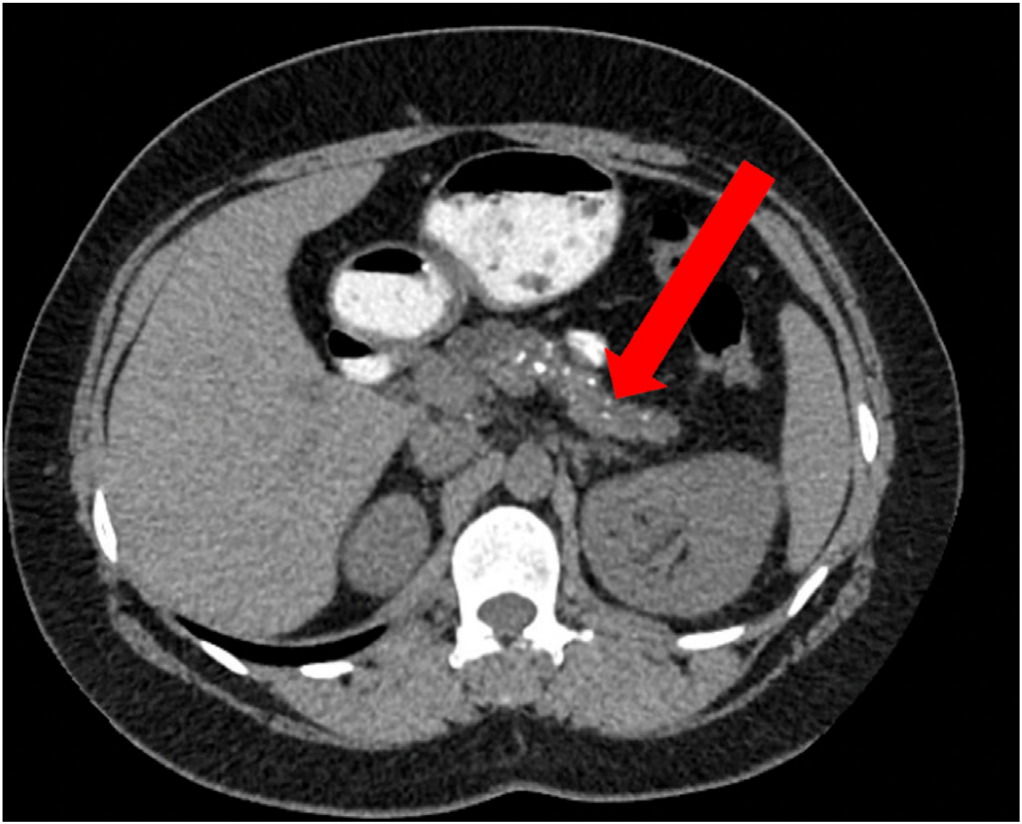
Computed tomography image showing an atrophic pancreas with multiple calcifications (*arrow*) consistent with chronic pancreatitis.

**Figure 6:**
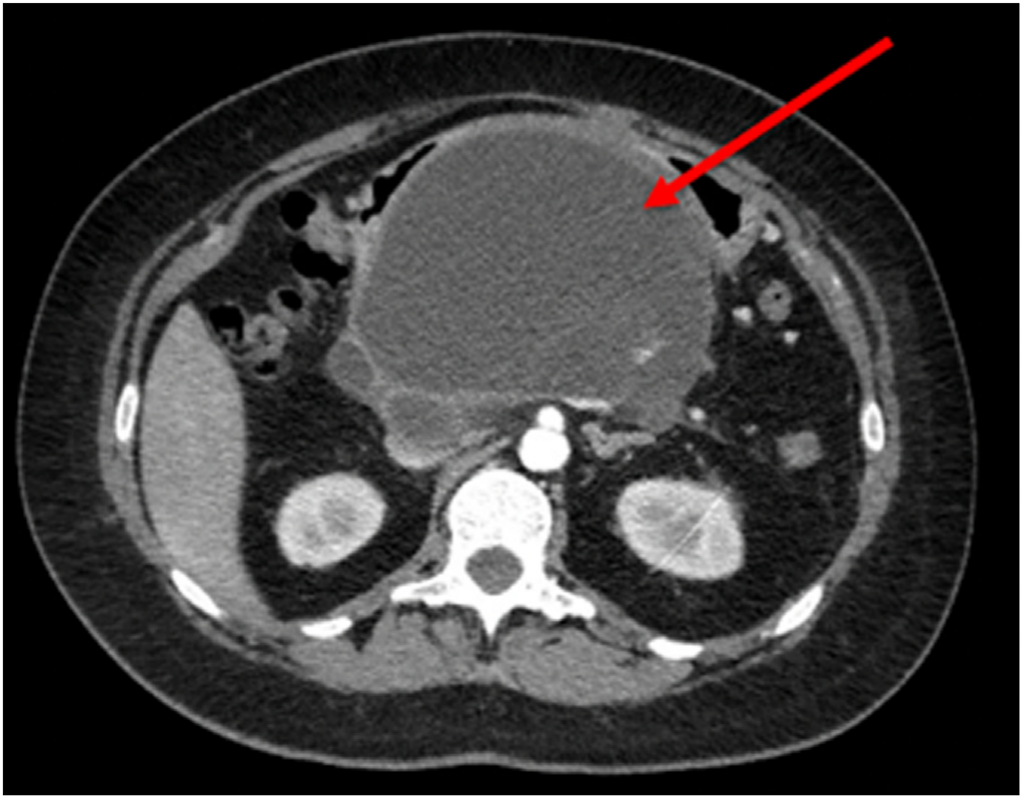
Computed tomography image demonstrating large pancreatic pseudocyst (*arrow*).

As the disease progresses, ductal obstruction resulting from pancreatic duct (PD) strictures and/or stones can occur (Fig. 4). Additionally, intraparenchymal hypertension can occur secondary to fibrosis and atrophy (Fig. 5). Adverse events from CP, such as pseudocysts, can also cause pain related to compression of adjacent organs (Fig. 6). Finally, there is evidence for pain hypersensitivity and central sensitization.^5^ These patients may have lower pain thresholds compared with other individuals without CP. A recent study showed that there are multiple pain-related factors in CP, including PD obstruction, abnormal pain processing, anxiety, depression, and pain catastrophizing. More than half of the study cohort had multiple factors, a cumulative effect was observed, and the number of factors presented had a significant impact on patient-reported outcomes.^6^

## Pain management

Many patients who experience abdominal pain secondary to CP require formal pain management plans in place. Owing to the multifactorial nature of chronic pain in CP, a multifaceted management strategy is typically required for optimal relief.

### Lifestyle modifications

Both alcohol use and tobacco use are associated with recurrent acute pancreatitis and the development of CP.^7^ Additionally, smoking increases the risk for the development of pancreatic ductal adenocarcinoma.^8^ All patients with CP should receive counseling on smoking and alcohol cessation.^7^ Referrals to self-help organizations such as Alcoholics Anonymous and referrals to mental health providers may be helpful. Additionally, pharmacologic therapy, including naltrexone, acamprosate, and disulfiram for alcohol abuse (and bupropion, varenicline, and nicotine replacement therapy for tobacco dependence) may improve success in this realm. Nonpharmacologic treatment such as cognitive behavioral therapy (CBT) may also help some motivated patients. APPs can play a critical role in counseling patients regarding smoking and alcohol cessation, providing ongoing support, and referring patients to appropriate resources.

### Pharmacologic therapy

**Figure 7:**
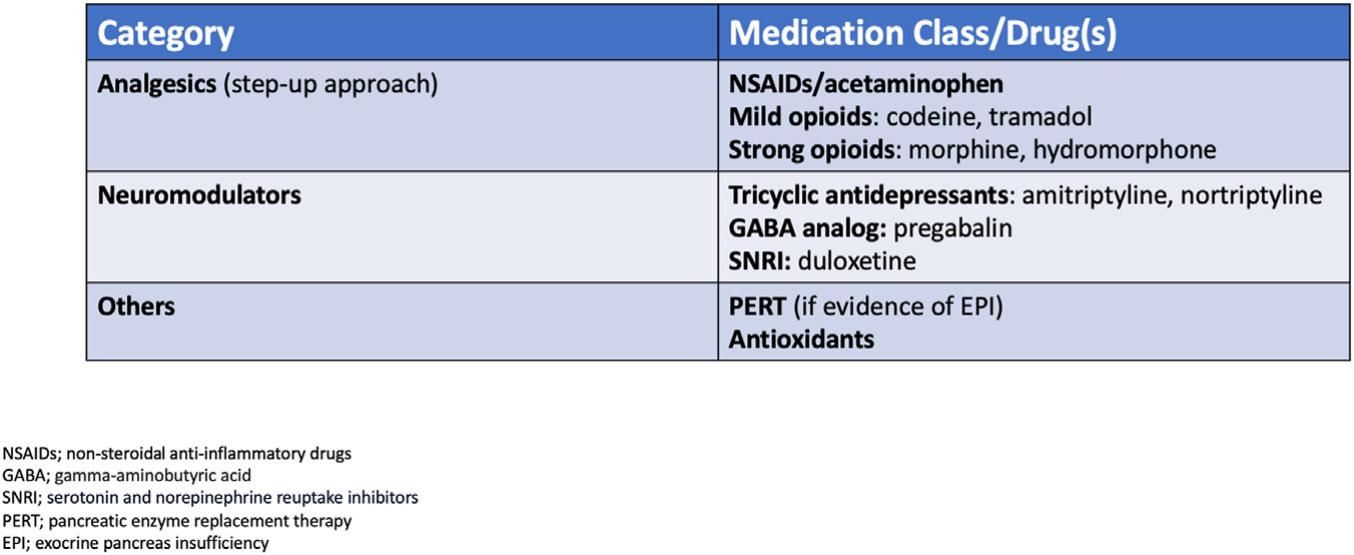
Pharmacologic therapy in chronic pancreatitis pain management.

Analgesics are often necessary for the management of pain in CP, particularly in patients who have acute chronic pancreatitis and those who have exhausted all other therapeutic options (Fig. 7). The World Health Organization Analgesic Ladder can be a helpful tool for providers, starting with nonsteroidal anti-inflammatory medications for mild pain and progressing to short-term opioids as needed for moderate to severe pain.^9^

Neuromodulators can be used to address alterations of central pain processing. Pregabalin has been shown to induce moderate pain relief in patients with CP.^10^ In a randomized controlled trial evaluating pregabalin versus placebo, pain relief after 3 weeks was achieved in statistically more patients who received pregabalin therapy (36% vs 24%, *P*=.02).^10^ Other options include tricyclic antidepressants (amitriptyline, nortriptyline) and serotonin-norepinephrine reuptake inhibitors (duloxetine).

There are insufficient data to recommend pancreatic enzyme replacement therapy for the management of pain in all CP patients. However, it is indicated in patients with evidence of exocrine pancreatic insufficiency.^7^ Patients with exocrine pancreatic insufficiency may experience postprandial abdominal cramping, bloating, steatorrhea, and weight loss. Although pancreatic enzyme replacement therapy is generally safe and well tolerated, its cost may be a barrier for some patients. Because of its safety profile, it can be considered a low-risk option for trying to improve pancreatitis-related pain.

In patients with increasing opioid requirements, systemic side effects such as constipation and opioid-related hyperalgesia can worsen the symptoms of abdominal pain. Intrathecal opioid pumps can be used as an alternative to decrease these systemic side effects.^11^^,^^12^

APPs can monitor medication adherence, assess side effects, and adjust treatment plans in collaboration with the care team. APPs may use tools such as medication logs and symptom tracking to evaluate adherence and effectiveness. By identifying potential issues early, such as opioid dependence, inadequate pain control, and adverse events, they can promptly adjust the therapeutic regimen and improve patients’ quality of life.

### Therapeutic endoscopy

Endoscopic therapy is warranted for patients who do not respond to conservative measures and in those with PD obstruction. For such patients, PD endotherapy with pancreatic sphincterotomy (with or without stone removal) and PD stenting can be performed.^13^ Pancreatic sphincterotomy has a technical success rate of 98%, but meaningful clinical success, defined as pain relief, varies widely. For example, in a randomized controlled trial, early surgical drainage outperformed endoscopic drainage, with only 32% of patients achieving complete or partial pain relief via endoscopy.^14^ Additionally, nearly 47% of patients who initially underwent endoscopic therapy eventually required surgery.^15^ Other studies, however, have reported partial or complete pain relief in ≤52% of patients.^16^, ^17^, ^18^ This suggests that whereas endoscopic approaches may not always provide durable or significant clinical benefit, there is still a subset of patients who experience sustained pain relief with endoscopic therapy alone. PD stenting, particularly for patients with main duct strictures or ductal disruption/leak, can reduce abdominal pain in approximately 85% of patients, although recurrence is common.^16^^,^^19^ The most common adverse events include acute pancreatitis, stent occlusion, and stent migration.^20^

In approximately 50% of patients with CP, PD calculi will develop. Standard ERCP techniques can be deployed to treat small PD calculi, but larger stones may require intraductal lithotripsy or extracorporeal shock wave lithotripsy.^21^ Lithotripsy is indicated in patients with recurrent pancreatic pain, moderate to marked changes in the pancreatic ductal system, and obstructing duct stones. More than 60% of patients achieve long-term pain relief after lithotripsy, although clinical outcomes may vary based on the extent of ductal disease.^16^^,^^22^

For patients with symptomatic pseudocysts, endoscopic ultrasound (EUS)-guided pseudocyst drainage has been shown to be effective in ≤95% of patients, with a recurrence rate of 10% to 20%.^23^^,^^24^

Celiac plexus block (CPB), involving local anesthetic and corticosteroid injection (bupivacaine and triamcinolone), can be performed under EUS guidance, provides temporary pain relief lasting 3 to 56 months, and can be repeated as needed.^7^^,^^25^ However, trials show that sequential CPB procedures may offer diminishing and less durable effects over time. Success rates are typically based on any degree of pain relief, and substantial variability exists in clinical response. CPB can also be performed with the use of percutaneous or surgical approaches.^7^

Abdominal pain in pancreatitis may worsen with oral feeding secondary to pancreatic stimulation and enzyme secretion. This may be minimized with jejunal feeding. Percutaneous endoscopic gastrostomy tubes with jejunal extension may be used to successfully improve nutrition indices and pain in patients with CP by decreasing this stimulus while simultaneously maintaining enteral nutrition.^26^ As with endoscopy procedures in general, APPs play a key role in periprocedural patient care for CP patients undergoing ERCP and EUS, including helping with management of anticoagulant/antiplatelet therapy, diabetes medications, postprocedural adverse events, follow-up care, and liaising with other specialties when such referrals are warranted.

### Surgical

Surgical interventions, including drainage procedures (Frey procedure, Puestow procedure) and resection (Whipple procedure, distal pancreatectomy, total pancreatectomy with islet cell transplantation), may play a pivotal role in addressing the underlying causes of pain in CP, especially in patients for whom medical therapy and endoscopic therapy have failed. By targeting the source of inflammation and obstruction, surgery offers high success rates for at least partial pain relief; however, the rates of adverse events can exceed 40% and may result in significant morbidity.

The ESCAPE randomized controlled trial demonstrated that early surgery can lead to significant pain relief with fewer total interventions compared with an endoscopy-first approach.^27^ At the 18-month follow-up visits, both cohorts had a similar proportion of patients with complete or partial pain relief, and there was no significant difference in pancreatic function or quality of life between the 2 groups.^5^^,^^7^^,^^27^ Although surgery has shown improved outcomes in pain relief compared with endoscopy, many patients often prefer a nonoperative approach first. The importance of a multidisciplinary approach in tailoring treatment strategies to the specific needs of patients, alleviating pain, and improving the overall quality of life cannot be overemphasized.

### Integrative medicine

**Figure 8:**
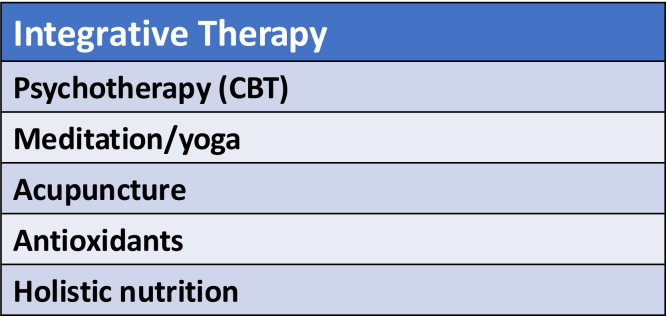
Integrative medicine for pain management in chronic pancreatitis. *CBT*, cognitive behavioral therapy.

The majority of reports related to pain management interventions for CP focus on pharmacologic and procedural (endoscopic/surgical) interventions. Integrative medicine is a novel approach to pain management and may be used as an alternative or adjunct method (Fig. 8).

CBT aims to reduce symptoms, including abdominal pain, depression, and anxiety, through psychosocial interventions. It is very effective in the treatment of substance abuse and may be beneficial for pain management in CP patients as well. A recent pilot study demonstrated patient acceptance and improvement in pain intensity and pain interference in patients who completed an internet-based CBT program compared with patients who received standard treatment regimens.^28^

Although data evaluating the use of acupuncture for the management of chronic pain in CP are very limited, acupuncture has been shown to be effective at reducing other chronic pain syndromes, including low back pain and headaches.^29^ A small randomized trial showed that acupuncture resulted in improved pain relief versus sham stimulation in patients with CP; however the effect was short lived, and long-term clinical data are lacking.^30^

Although medical cannabis may improve symptoms of pain in these patients, it has also been implicated as a possible cause of pancreatitis in some patients, so its use for symptom control cannot be universally recommended at this time.^31^^,^^32^

There are very limited data to suggest that antioxidants such as selenium, ascorbic acid, β-carotene, and methionine may improve pain in CP by reducing oxidative stress and exerting an anti-inflammatory effect.^33^^,^^34^ The optimal dosing and regimen are unclear, and these supplements are not regulated by the U.S. Food and Drug Administration.

## Challenges in Pain Management

There are several challenges in the management of pain in CP. Among the greatest are alcohol addiction, opioid addiction/dependence, and psychosocial issues. Patients actively using alcohol may be at higher risk for adverse events and decreased response from endoscopic or surgical interventions.^7^ Additionally, there is concern about providing opioids to patients with a history of, or risk for, substance abuse.

APPs play a pivotal role in addressing these challenges by providing consistent patient access, education, counseling, and support. They can identify patients at risk for substance abuse, facilitate referrals to addiction specialists, and work collaboratively within the care team to implement nonopioid pain management strategies. By maintaining close follow-up and monitoring, APPs ensure early intervention and may be able to improve adherence to treatment plans, improving patient outcomes.

Additional challenges include pain relapse, which may warrant escalating therapies, including more-invasive procedural and surgical interventions. The timing of therapeutic interventions is not always an easy decision, requiring an individualized approach and multidisciplinary discussion as well as shared decision making with the patient.

Providers must also keep in mind the potential for extrapancreatic pain and pain mimickers. Several other conditions such as gastroesophageal reflux disease, symptomatic pancreatic pseudocysts, duodenal obstruction, peptic ulcers, and bowel dysmotility may cause abdominal pain and confound the clinical picture in patients with CP.

## Summary

The approach to pain management in CP has evolved, emphasizing the need for a comprehensive and individualized strategy. Care should focus on identifying and addressing etiologic factors, mitigating lifestyle aggravators, and ruling out confounding conditions that may mimic CP symptoms. Pharmacotherapy remains an essential component but requires judicious use, including cautious opioid administration, trials of neuromodulators, and, when appropriate, the consideration of medical cannabis. The indications and long-term outcomes for endoscopic and surgical interventions are now better defined, providing clearer guidance for their use. Additionally, emerging approaches such as CBT and addressing the psychosocial aspects of care are increasingly recognized as integral to effective pain management.

APPs are uniquely positioned to enhance the treatment of CP patients by leveraging their expertise and patient-centered approach. By effectively integrating APPs into care teams, and providing them with mentorship, education, and specialized training, practices can optimize outcomes for patients with CP, as we have illustrated in this review. This collaborative and multidisciplinary approach fosters not only improved clinical results but also a more efficient and cohesive care environment and potential improvement in patient outcomes.

## Disclosure

All authors disclosed no financial relationships.
